# Resident-to-resident elder mistreatment (R-REM) intervention for direct care staff in assisted living residences: study protocol for a cluster randomized controlled trial

**DOI:** 10.1186/s13063-020-04580-z

**Published:** 2020-08-12

**Authors:** Jeanne A. Teresi, Stephanie Silver, Mildred Ramirez, Jian Kong, Joseph P. Eimicke, Gabriel D. Boratgis, Rhoda Meador, Leslie Schultz, Mark S. Lachs, Karl A. Pillemer

**Affiliations:** 1 Research Division, Hebrew Home at Riverdale, Bronx, NY USA; 2 Columbia University Stroud Center at New York State Psychiatric Institute, New York, NY USA; 3 Division of Geriatrics and Palliative Medicine, The Weill Medical College of Cornell University, New York, NY USA; 4 Bronfenbrenner Center for Translational Research, Cornell University, Ithaca, NY USA; 5 College of Human Ecology, Cornell University, Ithaca, NY USA

**Keywords:** Assisted living, Elder mistreatment, Randomized controlled trial

## Abstract

**Background:**

Resident-to-resident elder mistreatment (R-REM) is defined as negative and aggressive physical, sexual, or verbal interactions between (long-term care) residents that in a community setting would likely be construed as unwelcome and have high potential to cause physical and/or psychological harm and distress. R-REM has been established as a serious problem that has a negative impact on the safety, physical well-being, and quality-of-life of residents living in nursing homes. Although there are no in-depth studies, there is evidence that it is prevalent in assisted living residences and associated with a variety of person, environmental, and facility characteristics. The authors conducted the first systematic, prospective study of resident-to-resident elder mistreatment in nursing homes and developed an intervention for direct care staff to enhance knowledge of R-REM and increase reporting and resident safety by reducing falls and associated injuries. The study aim was to examine the effects of this intervention in assisted living residences. The primary distal outcome is falls and injuries, and the key process outcomes are staff knowledge and reporting.

**Methods:**

Twelve larger licensed assisted living residences with special care dementia units in two New York State regions will be enrolled on a rolling basis and randomized to intervention or usual care. Data derived from five sources, (1) resident interviews, (2) staff informants, (3) observational data, (4) chart, and (5) incident/accident report data, will be collected at baseline and 6 and 12 months with respect to 1050 residents (750 “downstate” and 300 “upstate”). The intervention is three training modules delivered on-site after baseline data collection for front line staff on all shifts in facilities randomized to the intervention. Modules relate to recognition, management, and reporting of resident-to-resident elder mistreatment.

**Discussion:**

Given the movement toward alternative congregate living arrangements for older individuals with significant comorbidities, including cognitive impairment; it is critical to enhance resident safety measured by falls, accidents, and injuries and staff knowledge related to recognition, reporting, and treatment of resident-to-resident aggressive and related negative interactions in such settings. This project is important in developing approaches for ameliorating and preventing R-REM in assisted living residences and enhancing resident safety and quality of life.

**Trial registration:**

ClinicalTrials.gov NCT03383289. Registered on 26 December 2017

## Administrative information

Note: the numbers in curly brackets in this protocol refer to SPIRIT checklist item numbers. The order of the items has been modified to group similar items (see http://www.equator-network.org/reporting-guidelines/spirit-2020-statement-defining-standard-protocol-items-for-clinical-trials/).

**Table:** 

Title {1}	Resident-to-Resident Elder Mistreatment (R-REM) Intervention for Direct Care Staff in Assisted Living Residences: Study Protocol for a Randomized Controlled Trial
Trial registration {2a and 2b}.	Clinical Trials.gov identifier: NCT03383289 registered December 26, 2017
Protocol version {3}	V1.0 December 26, 2017
Funding {4}	This study is supported by the National Institute on Aging (5R01AG057389).
Author details {5a}	1: Research Division, Hebrew Home at Riverdale, Bronx, NY, USA; 2: Columbia University Stroud Center at New York State Psychiatric Institute, New York, NY, USA; 3: Division of Geriatrics and Palliative Medicine, The Weill Medical College of Cornell University, New York, NY, USA; 4: Bronfenbrenner Center for Translational Research, Cornell University, Ithaca, NY, USA 5: College of Human Ecology, Cornell University, Ithaca, NY, USA
Name and contact information for the trial sponsor {5b}	the National Institute on Aging (NIA)
Role of sponsor {5c}	The NIA has no role or authority in the study design; collection, management, analysis, and interpretation of data; writing of the report; and the decision to submit the report for publication.

## Introduction

### Background and rationale {6a}

Researchers have recently begun to address negative and aggressive interactions among residents in long-term care. However, scientific attention is increasing [1–7] because such aggression has been found to be extensive and has the potential to impact physically and/or cause psychological distress to both residents and staff [8, 9]. A few studies discussing elder abuse in the residential care sector address resident-to-resident elder mistreatment (R-REM) in assisted living residences (ALRs) [10–12]. Benson [13] examined “relational aggression,” defined as a manipulative, non-physical form of aggression using rumor or gossip. An extensive study on elder abuse in residential care facilities [14] highlights the need for staff training and behavior management strategies to counter serious outcomes such as physical injury and emotional distress. Similarly, Caspi [15], examining R-REM in two special care units of an ALR, identified staff prevention strategies and suggested incorporating those into care staff training programs. Castle [16] reported the perceptions about elder abuse among administrators and direct care workers in 1500 randomly selected ALRs nationwide, documenting that R-REM was reported to be more common than staff-to-resident abuse*.* A recent study [17] reported the 30-day prevalence of physical (7.6%), verbal (9.5%), and sexual (2.0%) aggression by ALR residents toward other residents or staff based on data from the 2010 National Survey of Residential Care Facilities. They found a five, four, and twofold increase, respectively in the likelihood of engaging in these behaviors for residents with Alzheimer’s disease and related disorders. These authors call for training and prevention in ALR. Thus, R-REM in residential care in general, and specifically in AL settings, although prevalent, has received little attention.

The authors conducted the first systematic, prospective study of resident-to-resident elder mistreatment (R-REM) in nursing homes and have developed novel methodology to identify the phenomenon. Data demonstrated that (1) R-REM is highly prevalent, (2) case finding methodology is greatly enhanced by meaningful participation of front line staff, and (3) paradoxically, higher-functioning residents may be at greatest risk for involvement in R-REM (e.g., residents who are ambulatory and capable of wandering as opposed to those who are in the last stages of dementing illness). Additionally, funded by the New York State Department of Health dementia grants program, we developed a three-module program targeting front line staff to implement best practices related to R-REM in long-term services and support (LTSS) settings. Using an experimental design, we tested the intervention in nursing homes. Evaluation of longitudinal outcomes showed significant increases in staff knowledge post training, controlling for pre-training levels for the intervention group and of increased recognition of R-REM, and longitudinal reporting in the intervention as contrasted with the usual care group [7]. Additionally, falls, accidents, and injuries were reduced [18]. These findings have important implications for assisted living residences (ALRs) where residents typically have cognitive impairment with better mobility and less staff to intervene in R-REM. The state survey process is also less uniform in ALRs (in comparison to nursing homes) and has not addressed R-REM.

### Objectives {7}

The goal of the project is to evaluate a training program for staff that enhances identification and intervention with respect to episodes of resident-to-resident elder mistreatment (R-REM). The hypotheses corresponding to the specific process outcome aims (1 and 2) and primary distal outcome (aim 3) for which the study was powered are:

- *Aim 1 (A1)*. Enhance staff knowledge of R-REM
  - *Hypothesis 1*: Staff knowledge related to R-REM and R-REM treatment will increase after training.
- *Aim 2 (A2)*. Enhance staff recognition, reporting, and care planning related to R-REM
  - *Hypothesis 2*: Due to the heightened awareness as a result of training, the frequency of reported R-REM in the intervention group will increase, relative to the comparison group after training.
- *Aim 3 (A3)*. Evaluate the impact of the staff intervention on resident falls, accidents, and injuries and on quality of life using a prospective experimental design that derives information from five sources: (1) resident interviews, (2) staff informants, (3) observational data, (4) chart, and (5) incident/accident report data.
  - *Primary hypothesis*: The frequency of falls, accidents, and injuries will decrease in the intervention group, relative to the comparison group after implementation of the training intervention.
  - *Secondary hypotheses*:
    1. Resident quality of life as measured by affective state will improve in the intervention group, relative to the comparison group after implementation of the training intervention.
    2. Resident behavior problems will decline in the intervention group, relative to the comparison group after implementation of the training intervention. Resident behaviors will mediate the relationship between the intervention and the falls/accidents/injuries outcome.

### Trial design {8}

This is a pragmatic, prospective, cluster randomized trial (facility level) design with three waves of data collection (baseline, 6- and 12-months) at 12 assisted living facilities in two New York regions. There will be three levels of clustering: facilities within regions, units within facilities and repeated measures on residents within units, with the regions fixed. On a rolling basis, six facilities will be selected at random and recruited in New York City and the nearby suburban counties (downstate region). Six facilities in the upstate New York region of Rochester will also be randomly selected. Three facilities in each region will be randomized to usual care or to the intervention on a 1:1 ratio. Facilities allocated to the usual care group will receive the intervention after completion of their 12-month data collection.

## Methods: participants, interventions, and outcomes

### Study setting {9}

Because an aim of the study was to examine R-REM in all residents, including those with Alzheimer’s disease, to maximize resources, we restricted our sample to larger licensed ALRs with special needs (including memory care) units in the two selected New York regions. The sample includes facilities with special needs units because the likelihood of R-REM is greater there [2]. Upstate, we selected from the population of 33 facilities with bed sizes of 50 and over with special units for individuals with cognitive impairment. Downstate, there are 50 larger (80+ bed) facilities with special needs units for cognitive impairment in the selected area.

### Eligibility criteria {10}

Because of the longitudinal nature of the study, it was desirable to screen out short-stay residents. *All residents* on long-term care units, except residents receiving hospice care in the sampled facilities, will be invited to participate.

Facilities will have the option to exclude individuals for selected reasons. For residents unable to complete the consent process (due to, e.g., cognitive impairment, language barrier, health impairment), consent will be sought by designated proxies (families or legal guardians). Residents unable to respond (due to language other than English or Spanish, or impairment) will be excluded from resident-level measures; chart review, staff informant, and observational measures will be performed on those whose families provide proxy consent.

### Who will take informed consent? {26a}

A formal “Informed consent and HIPAA Authorization” document was approved by the Weill Cornell Medicine Institutional Review Board (IRB). A brief screen is used to determine capacity to provide informed consent. This procedure has been used in several large studies of nursing homes and assisted living residents and has been approved by several IRBs. (Family members of individuals who are unable to provide informed consent will be contacted to obtain consent.) Additionally, family members of all residents will be sent letters informing them of the study with an opt-out option.

The actual informed consent is obtained after the resident either (a) reads the Informed Consent Form or (b) the interviewer reads the Informed Consent Form to the resident.

There are several cases where a verbal informed consent is used in place of a written informed consent:

1. If the resident has a perceptual impairment that makes it difficult for her/him to read/sign the Informed Consent Form (e.g., visual impairment)
2. If the resident is illiterate, the interviewer reads the Informed Consent Form to the resident
3. If the resident has a physical impairment that prevents her/him from writing (e.g., contractures in both arms, paralysis, etc.)
4. If the resident does not wish to sign the Informed Consent Form, but verbally indicates that s/he is willing to participate in the interview

Per the IRB, in these cases, an observer not associated with the research (e.g., facility staff) must witness the verbal consent process.

### Additional consent provisions for collection and use of participant data and biological specimens {26b}

Not applicable, no specimens collected.

## Interventions

### Explanation for the choice of comparators {6b}

After successful implementation of the R-REM research protocol in nursing homes, the authors wanted to further test the intervention in another LTSS setting with a similar population but with less staffing and state oversight. Assisted living was selected because it is an area of LTSS that is growing rapidly both in numbers of facilities and in terms of the populations served.

### Intervention description {11a}

The intervention, i.e., the training of nursing, social work, administrative, and other staff on R-REM is conducted in three separate sessions: (1) recognition and risk factors, (2) management, and (3) implementation of guidelines. The trainer who administers the three modules is an experienced doctoral-level, adult education professional who participated in the development and modification of the modules; she has extensive experience in staff training. Modules are provided to facilities randomized to the intervention after the completion of baseline data collection and to those randomized to usual care after completion of 12-month follow-up data collection.

#### Description of module 1: recognizing R-REM

Module 1 covers the extent of R-REM which includes evidence; risk factors associated with the victims, perpetrators, and environment; and the role of cognitive impairment. Different forms of mistreatment are covered, including physical, psychological, sexual, and theft. This module is delivered in the form of an experiential half-hour in-service training, plus pre- and post-tests designed to be conducted at the ALRs.

#### Description of module 2: management of R-REM

A. Introduction and review of previous session and pretest
B. Film on management of elder mistreatment
C. A presentation of the SEARCH (Support, Evaluate, Act, Report, Care Plan, Help to Avoid) approach to R-REM management; review, lessons learned; post-test.

The 25-min film for this module was designed by the research team and directed and produced by the New York University Department of Media Production. It was narrated by distinguished journalist, Charles Osgood, and includes a discussion of what constitutes putative evidence of serious abuse, such as bruises, cuts, or more serious injuries (broken bones or cracked ribs). Mistreatment such as verbal aggression and threats, sexual harassment, and missing belongings are discussed. Three skits by professional actors are presented: *skit 1*, most obvious form of elder mistreatment: physical assault; *skit 2*, less obvious form of elder mistreatment: verbal insult; and *skit 3*, subtle form of elder mistreatment: psychological abuse, e.g., wandering uninvited into another’s room and rummaging through another resident’s property.

Each skit is followed by an example of a poor staff response to the event as well as a better practice, and by commentaries by leading multidisciplinary experts in elder abuse, representing different perspectives: psycho-social, medical, nursing, administrator/legal.

The final component of the video is a review of nine steps to manage and curb R-REM.

#### Description of module 3: implementation of best practices related to R-REM

1. Introduction and review of previous session;
2. Presentation of implementation methods and forms (The R-REM Behavior Recognition and Documentation Sheet -- BRDS); discussion of methods for completion;
3. Presentation of filmed vignettes for practice in the completion of the BRDS;
4. Review practice sheets and lessons learned;
5. Review of implementation guidelines.

The focus of this session is on the intervention fidelity and implementation measures, including implementation of reporting guidelines. The training includes video vignettes that are rated and reviewed to confirm skills. Ways to enhance positive group relationships and the use of community to counteract individual acts of mistreatment are discussed, addressing the question, how can staff work together to structure the social and physical environment to mitigate R-REM? The importance and rules for reporting R-REM are reviewed.

### Criteria for discontinuing or modifying allocated interventions {11b}

There are no predetermined criteria for discontinuing or modifying the intervention.

### Strategies to improve adherence to interventions {11c}

**Fig. 1:**
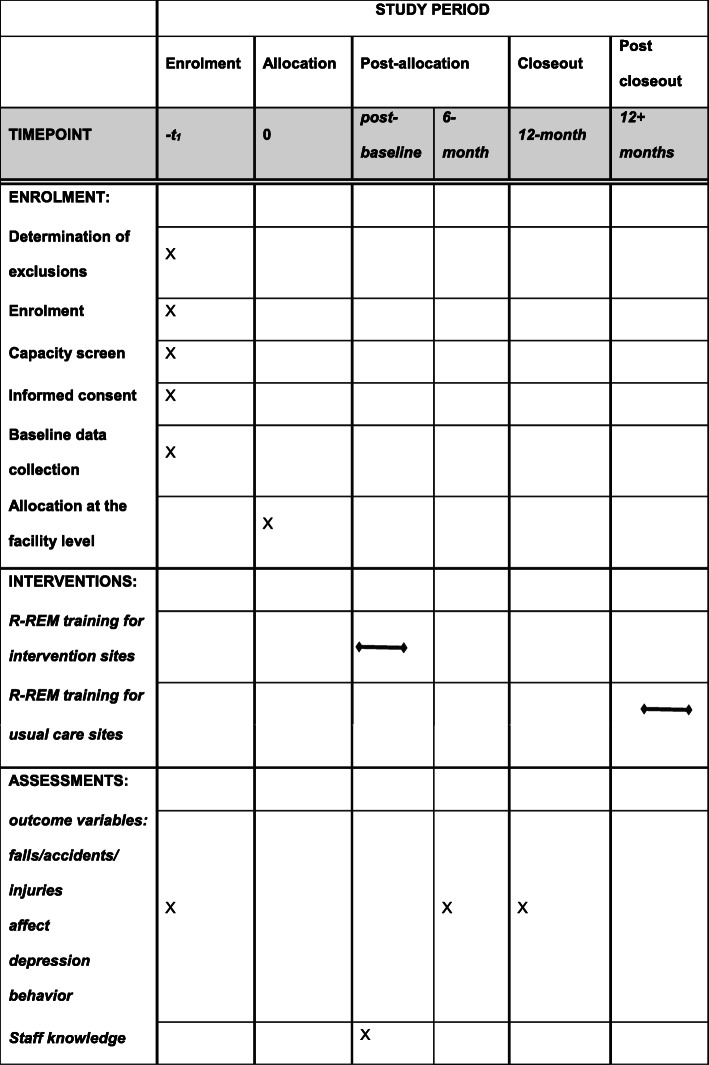
Schedule of enrolment, interventions, and assessments

Each session is scheduled twice for all shifts, including the night staff (Fig. 1). Additional makeup sessions are available for those staff unable to attend the original training sessions. Facility administrators alert the staff of the mandatory training sessions using several methods including flyers posted in the staff room and notices in pay stubs.

### Relevant concomitant care permitted or prohibited during the trial {11d}

None

### Provisions for post-trial care {30}

There are no such provisions for this non-invasive minimal risk intervention for ALR staff.

### Outcomes {12}

#### Aim 1: Process level 2 evaluation of staff knowledge outcomes: knowledge tests

Ten question pre-post knowledge tests were developed for each of the first two R-REM training modules, i.e., Recognition and Risk Factors, and Management (the SEARCH approach) based on its respective content. An additional knowledge test was developed for the third module, Implementation Guidelines, in order to assess use of the BRDS to document R-REM events. The latter test compares staff answers to the gold standard ratings. Hypothesis 1, regarding enhanced staff knowledge, will be examined for individual knowledge items using paired *t* tests, comparing pre–post knowledge scores between groups, adjusting standard errors for clustered data within facilities. Group differences in total scores will be examined using a linear mixed (fixed and random effects) model for effect estimation.

#### Aim 2: Process level 3 evaluation of implementation outcomes: recognition and reporting using the R-REM measures

##### R-REM behavior recognition and documentation sheet (BRDS)

This measure was based on the Shift Coupon, a form originally designed by nursing staff to provide a “quick, easy, anonymous, and non-threatening method to report adverse events” [19], and is in the form of a small note pad. BRDSs are intended to measure recognition and capture real time R-REM events. Hypothesis 2, regarding enhanced R-REM recognition, will be examined with a chi-square analysis comparing counts of BRDS reports from experimental and comparison facilities captured over time and treated as binary incidents. Reporting implementation will be examined by evaluating the differences between experimental and comparison group staff reports of R-REM, collected over three waves. Individual R-REM reporting will be determined by counting whether any of the staff-reported R-REM indicators are positive. The object of this analysis is not to identify individuals and perpetrators, but to examine the reported events. In modeling R-REM events, a Poisson regression of count data will be performed.

#### Aim 3: Primary level-4 evaluation of distal outcomes: summary of the sources of data for determining falls/accidents/injuries and incidents of R-REM

The data used for evaluation of the primary distal outcome and for R-REM reports include (a) self-report among those residents who are capable, (b) accident and incident reports, (c) resident record review, (d) observational data, including the BRDS, and (e) reports from staff. In addition, an environmental evaluation will occur. Data for falls and injuries will be collected via chart review, staff report and residents who can self-report, and from Incident/Accident Reports on an ongoing basis. Resident and staff informant information will be collected at baseline and 6- and 12-month follow-up. Falls will be considered as binomial (any fall) and Poisson distributed (fall counts).

### Participant timeline {13}

There will be rolling enrollment of ALRs. As each facility is enrolled, the full census is obtained. Those on short stay units, those on hospice, and those who the facility deems ineligible to participate are removed from the list. All remaining residents are approached for participation and baseline data collection occurs. For those unable to provide consent, family consent is sought. Once all residents of the facility are recruited and baseline data collection is completed, the facility is randomized to the usual care or intervention. Intervention facility staff then receives training in all three modules. The research team returns for 6-month and 12-month data collection.

### Sample size {14}

Power calculations are provided for the primary distal outcome: falls, including accidents and injuries, also the outcome requiring the largest sample size. Upstate, it is expected that an average of 50 residents per site will be selected for a total of 300. Downstate, the facilities are larger, and it is expected that the average size will be 125 residents or 750 total. The proposed sample size is 6 facilities and 525 residents per arm.

Using the canonical link (Logit), the generalized linear model (GLIMMIX):*η* = log(*p*_ijk_/(1 − *p*_ijk_)) = *β*_0_ + *β*_1_*X*_ijk_ + FU_jk_, where FU_jk_ is a random effect associated with facility and unit.

The formula for the sample size per group using the method of Diggle is: ${m}^{\ast }={\left({z}_{\alpha}\sqrt{2\overline{P}\overline{Q}}+{z}_{\beta}\sqrt{p_0{q}_0+{p}_1{q}_1}\right)}^2\left(1+\left(n-1\right)\rho \right)/\left(n{\left({p}_1-{p}_0\right)}^2\right)$; and using the GEE method: ${m}^{\ast }=\frac{{\left({Z}_{1-\alpha /2}+{Z}_{1-\beta}\right)}^2\left({\pi}_1{p}_0\left(1-{p}_0\right)+{\pi}_0{p}_1\left(1-{p}_1\right)\right)\left(1+\left(n-1\right)\rho \right)}{2n{\pi}_0{\pi}_1{\left({p}_0-{p}_1\right)}^2}$. The formula below is adjusted with variance inflation factor (*V*_if_) and reliability (*R*_el_): *m = V*_if_m*/*R*_*el*_ and

$$ {V}_{\mathrm{if}}=\left(1+\left({n}_e-1\right){\rho}_e\right)\left(1+\left({n}_s-1\right){\rho}_s{n}_e{p}_e/\left(1+\left({n}_e-1\right){\rho}_e\right)\right) $$

where *n*_s_ = 5 is the average number of units in the facility, *ρ*_s_ = 0.015 is the ICC for facility, *n*_e_ = 17 is the average number of residents in the unit, *ρ*_e_ = 0.03 is the ICC for unit, and the reliability is *R*_el_ = 0.95.

The following table assumes *α* = 0.05, power (1 − β) = 80%, *R* = 0.95, and *V*_if_ = 2.5 (with n_e_ = 17, ICC_Unit_ = 0.03, *N*_s_ = 5, ICC_Facility_ = 0.015, the fall rate at follow-up: *p*_0_ = 33% and *p*_1_ = 20.75%). Two scenarios for *ρ* (the average correlation of the outcomes over waves) were posited:

**Table:** 

Group	Fall rate *P*(*y* = 1)				Diggle method		GEE method	
	Baseline rate (%)	6 months (%)	12 months (%)	Combined 6 months and 12 months (%)	M (*ρ* = 0.5)	M (*ρ* = 0.6)	M (*ρ* = 0.5)	M (*ρ* = 0.6)
Inter-vention	37	22	19.5	20.75	404	431	399	425
Usual care	37	34	32	33	404	431	399	425

As shown, with 525 per group, power is adequate for intent-to-treat (ITT) analyses, including all respondents. Because randomization is at the facility rather than individual level, there is the potential for imbalance on baseline variables and missing covariate data. Assuming attrition of 15% at wave 1, it is estimated that 445 per group will have at least baseline and one additional wave of data included, using the EM missing data algorithm; thus, power is adequate to detect the posited difference in fall reduction of about 12% even with attrition.

Although we did not include power calculations for the process level 2 and level 3 evaluation outcomes in the protocol, a brief summary of these analyses is provided. As shown, the sample size requirement for these outcomes is less than that of the primary distal outcome of falls, including injuries for which the power calculations were provided in the study protocol.

With respect to the level 2 knowledge outcome, in an earlier study [7] of 270 to 340 staff, we performed a paired *t* test using a mixed model analyses adjusting for clustering within facilities and covariates as needed. The effect size across primary and sensitivity analyses (which were highly significant; *p* < 0.001) was between − 0.696 and − 0.964, indicating that a minimally detectable effect size was a one-point or less improvement on a 10-item knowledge test. In the current study, we calculated power for the knowledge test under reasonable scenarios observed in previous studies. For *ρ* = 0.60 (the correlation between pre and post-tests), a small effect size (Cohen’s *d* = 0.20, 0.24 points assuming *σ* = 1.2, or 0.30 points assuming *σ* = 1.5) is detectable. As shown, a very small effect size (less than one point on a 10-point knowledge test) is detectable, given the anticipated sample size of staff.

With respect to the level 3 process evaluation outcome of increased reporting, in a previous study [7] in another setting, it was observed that the intervention group reported significantly more incidents after implementation of the intervention than the usual care group. A Poisson regression (generalized multivariate linear model; GML) with a log link will be used to model the incident event counts in the current study. Our previous analyses showed an annual prevalence of R-REM of 25%. Assuming that the usual care and intervention group start at about the same level, power was calculated to detect clinically important differential rates of R-REM reporting. The assumptions were as follows: *α* = 0.05, 1-β = 0.80, *R*^2^ = 0.16 (adjusted for multivariate covariates), *R*_e_ (reliability) = 0.95, and variance inflation factor (*V*_IF_) = 1.18. The results show that given the proposed sample size, it is possible to detect differential R-REM reporting incidence rates of *λ*_0_ = 0.25 in the usual care group and *λ*_1_ (intervention group) = 0.40 for the GML method and 0.385 for sensitivity analyses using the exact score method. An incidence rate of (*λ*_0_) = 0.25 translates to *π*_0_ = 1−e^−λ0^ = 22.1% (the proportion reporting R-REM in the usual care group), and (*λ*_1_) = 0.40 translates to *π*_1_ = 1−e^−λ1^ = 33.0% (the proportion of R-REM reports among the intervention group). With a total *N* = 800 (400 patients per group), power is 80% to detect differences in reporting as small as 10 to 11% with a two-sided test (*α* = 0.05), adjusted for multivariate covariates, unreliability, and clustering. This difference is smaller than that observed in previous studies.

### Recruitment {15}

All residents are approached and invited to participate. For those who do not wish to be interviewed, a different or the same interviewer approaches again at another time that may be more amenable to the resident. All resident questions are addressed and interviews occur at a time and place preferred by the resident.

## Assignment of interventions: allocation

### Sequence generation {16a}

Algorithms for conducting randomization that take into account clustering such as exists with geographical regions and rolling enrollment have been developed by the author. This method has yielded balanced groups for many studies. This randomization procedure will be carried out using a SAS macro after a facility completes the baseline interview. A random number from 0 to 1 will be used to determine the assignment group. The standard cut score will be set at 0.5 for the first n facilities from the same region. Facilities which receive a random number between 0 and 0.5 will be assigned to the usual care group and those with a random number greater than 0.5 will be assigned to the intervention group. The balance between the groups within each group will be carefully weighted after the total number of facilities from a group reaches a number greater than *n*. Before the randomization procedure, the number of facilities randomized to each arm for each region will be estimated using SAS macro programs. If more than the *n* facilities are randomized initially, the cut score for the next facility is equal to the ratio of the intervention group (*n*1) to the facilities already randomized (*m*) for that group (*n*1/*m*). For example, group A region provides eight facilities for randomization and the *n* is set to 5, the first five facilities (*n*) will be randomized to the standard cut score 0.5 (about half will go to the intervention and half to the usual care group). The sixth facility’s randomization cut score is equal to the number of facilities in the intervention group (*n*1) divided by the total number of facilities randomized within that group (in this case, the denominator *m* is 5.) The seventh facility’s cut score will be adjusted according to the previous six facilities, and so forth.

### Concealment mechanism {16b}

The biostatistician who runs the randomization procedure passes the information directly to the intervention trainer who alerts the ALRs of their assignment. The principal investigator who oversees the data coordinating center (DCC) at the Research Division, Hebrew Home at Riverdale (RD-HHAR), is the only other person made aware of the assignment.

### Implementation {16c}

A biostatistician at the coordinating center developed and runs the randomization procedure. Randomization occurs at the facility level. Research interviewers who enroll participants are blinded to randomization group.

## Assignment of interventions: blinding

### Who will be blinded {17a}

Research staff who interact with facilities are blinded to the extent possible; project managers, field managers, and research interviewers are not made aware of the site assignment. ALR residents are not made aware of group assignment. It is possible that ALR staff in facilities assigned to the intervention may mention the training to research staff at either the 6- or 12-month follow-up data collection.

### Procedure for unblinding if needed {17b}

Not applicable, there are no circumstances where unblinding would be needed.

## Data collection and management

### Plans for assessment and collection of outcomes {18a}

Data for aim 1: ten question pre-post knowledge tests were developed for each of the first two R-REM training modules. Data for aims 2 and 3 are derived from five sources: (1) resident interviews, (2) staff informants, (3) observational data, (4) chart, and (5) incident/accident report data. Research interviewers attend a 5-day training session that includes how to administer questionnaires. Training continues in the field.

#### Resident interview

##### Determination of capacity to provide informed consent and participate

A brief screen will be used to determine capacity to provide informed consent (see Protection of Human Subjects).

##### Institutional Comprehensive Assessment and Referral Evaluation (INCARE) [20–22]

Individuals who are able to provide informed consent (or whose family members consent on their behalf) will be assessed with the INCARE, a multilevel-multi source instrument that contains quality of life outcomes and case-mix covariates and allows at least some assessment to be completed across all levels of residents. It includes (a) arousal and cognitive functioning (orientation, memory, calculation/attention), (b) range of motion and ambulation, (c) affect, and (d) behavior. It includes the Care Dementia Diagnostic Scale (CAREDIAG) which has been studied using several advanced psychometric models, including analyses of its relationship to dementia diagnosis [23, 24]. Scales for each construct have evidenced moderate to high (Cronbach’s alpha) reliability coefficients. Estimates for the ADL scales range from 0.59 for standing disability to 0.95 for total ADL/ambulation disorder, from 0.86 to 0.94 for the cognitive scales, and 0.90’s for the behavior scales [25]. The McDonald’s omega was estimated at 0.95 for the CAREDIAG.

##### The Feeling Tone Questionnaire (FTQ)

The FTQ measure of affect contains 16 questions asked of the resident. Typical items are “Are you feeling well?”, “Are you feeling happy today?”, and “Do you feel lonely?”. Each item is coded “yes,” “no,” or “equivocal (sometimes, it depends),” and the response rated for affect using a 5-point continuum from 1—“laughs, praises, enthusiastic, emphatically positive”—to 5—“extreme negative—cries, groans, curses, is emphatically negative.” Three scales are scored: response, affect, and total. The FTQ has been used among numerous samples of nursing home residents, in which reliabilities were in the 0.90’s. In an AL sample, alphas ranged from 0.73 to 0.90 at baseline and from .77 to 0.93 at follow-up. The measure has recently been evaluated and shortened [26] using a large sample of 6000 LTSS residents, including AL.

##### Performance Activities of Daily Living (PADL)

The PADL [27] (alpha typically in the 0.90’s) [28] is a 27-item scale that measures an individual’s lack of ability to perform various upper and lower body movement tasks associated with eating, dressing, and grooming, such as putting on a sweater, buttoning and unbuttoning a sweater, guiding a spoon to the mouth, and combing hair independently. Performance times are recorded, and items are rated as to whether the task was performed with or without cueing, or could not be performed at all.

##### Extended interview

An extended interview will be administered to participants with sufficient cognitive abilities to respond. Research has demonstrated that many residents can reliably self-report yes/no questions about daily care [29], life satisfaction [30], pain [31], and quality of life [32–34]. It is anticipated that about 70 to 80% of residents will be included at this stage. This interview includes scales of fear of falling/falls history, depression/affect, and ADLs [35]. The number of falls experienced in the past year and the Fear of Falling scale, comprising eight items tapping feelings regarding fear of falling and the reasons for fear, will be administered. The alpha coefficient for this scale for a prior NY State Assisted Living sample was .84 at baseline and 0.85 at follow-up.

##### Depression scales

The Short Care [21, 22, 36, 37] (alpha of 0.83 for an assisted living sample at baseline and 0.81 at follow-up) typical items are “feeling sad or depressed during the past month,” “cried during the past month”, and “lie awake at night with depressed thoughts.” Included is an anchored four-point global rating of happiness level. We will also include the Patient Reported Outcomes Measurement Information System (PROMIS) Depression scale [38].

##### Resident-to-resident elder mistreatment—resident version (R-REM-R)

For residents who are capable, the R-REM-R will be used to determine whether an incident of R-REM has been directed at the respondent. This measure was created by the applicant team by combining desirable aspects of the most commonly used instrument in violence research [39] with a measure used to rate behavioral disturbance in LTSS [8]. The measure contains 22 items related to verbal, physical, sexual, and other behaviors along with their frequency during the 2 weeks and the past year. After focus groups with LTSS staff, more R-REM items were added and a follow-up distress question was added to all endorsed items. The Cronbach’s alpha estimate for the scale was 0.90.

#### Staff informant interview

A brief informant interview will be administered to the staff member most familiar with the participant.

##### Nurse informant rating of behaviors

The Nurse/Primary Care Worker Informant Interview [40] will be used. Typical items include the following: “wanders during the day,” “repetitive questioning,” “argumentative,” “demanding,” and “disrupts other’s activities”. Items are rated for frequency: “not at all,” “sometimes (1–4 times per week),” and “often (5+ times per week).” The Cronbach’s alpha estimate was in the 0.80s, and the 0.60s in an urban and rural LTSS sample [41].

##### The R-REM Staff Version

Was developed along with the resident version (see above). R-REM is operationalized as *staff* endorsing (or incident reports of) any items on the R-REM interview. The following instructions are given to staff: “We are trying to find out about things residents have done to other residents. I’d like you to think about incidents involving (resident) and one or more people living here. We’ll focus on different forms of resident-to-resident mistreatment. This can include verbal incidents like: residents saying mean things to each other, insulting each other’s race or ethnic group, and/or screaming at each other. Physical incidents can include: hitting, pushing, and/or grabbing. Sexual incidents may include touching, or saying or doing sexual things that made other residents feel uncomfortable. We are also interested in incidents involving other residents going into rooms without being asked, touching personal things, or throwing things. We are referring to both serious reportable and minor incidents that would not necessarily be formally reported. Remember we are talking about incidents in the past two weeks that involved (resident).” A list of R-REM behaviors is provided. Location, time, the identity of the person who started the incident, and a description of other participant(s) (sex and relationship) are recorded. The staff member reports what s/he did about this (e.g., separated or redirected residents). The Cronbach’s alpha estimate for the R-REM scale was 0.90. The Schmid-Leiman bi-factor model identified three group factors: verbal, physical, and a less differentiated factor including items on room invasion, throwing, and threatening gestures. An additional item related to sexual encounters was also included [4, 42]. The alpha estimate from the “psych” R package [43, 44] was 0.94, omega hierarchical 0.76, omega total 0.97, and explained common variance (ECV) [45] was 0.59.

#### Observational data

A. For each participant, ten 5-min observations are performed by trained interviewers at different times of the day and in different locations in order to obtain additional information about incidents of R-REM and other disturbed behaviors. While episodes of R-REM are likely to be brief and intermittent, based on our experience in the nursing home study, interviewers did observe many incidents of R-REM. The Total Observation Checklist includes 14 affect and 37 behavior items (Observed Behavior Checklist) [43]. Frequencies of affective and behavioral states are coded as “not at all,” “very little (1 or 2xs during observation period),” “with some frequency (several times),” “with moderate frequency (many times but not continuous),” or “with great frequency (continuous).” Typical affect items include “crying,” “agitated,” and “emotionally labile.” Typical behavioral items include “disruptive of others,” “wandering,” and “argumentative.” In an urban LTSS sample, the Cronbach’s alphas ranged from .74 to 0.85 and from 0.85 to 0.87 in a rural LTSS sample [43].
B. *R-REM behavior recognition and documentation sheet (BRDS)*: This measure was based on the Shift Coupon, a form originally designed by nursing staff to provide a “quick, easy, anonymous, and non-threatening method to report adverse events” [19], and was in the form of a small note pad. BRDSs are intended to measure recognition and capture real time R-REM events. These forms are designed as prescription pads to be carried in the pockets of staff. They are distributed at the training sessions (module 3 for intervention facilities and during the opening of the unit for usual care staff); additional pads are available at a central location. Sheets are torn off after documenting R-REM. Items include residents involved, identity of the perpetrator, actions involved, location, potential cause, and what did you (staff) do about it. Boxes for completed forms are placed in a designated location on each unit.
C. *Event logs*: When an event is reported from (a) resident interview, (b) staff interview, (c) interviewer observation, or (d) BRDS, the research interviewer will complete an Event Log worksheet in order to better understand the circumstances of the R-REM event. This form contains descriptive information about the event, its time and place, the reporting source, the participants, and environmental factors at the time of the event.

#### Chart data

Demographic variables of interest include age, race, educational attainment, and length of stay in the facility. Medical diagnoses, medications, activities of daily living, cognitive impairment, and mental health data will be extracted from the Assisted Living Residence Medical Evaluation form. Diagnosis information will be integrated into a comorbidity index, in order to adjust for burden of chronic illnesses. Interrater reliability will be examined on a random subset of charts. In addition, physical function, including history of falls in the past 3 months, frequency of falls, resulting injuries, and open-ended comments and behavioral issues data will be extracted from the Assisted Living Resident Evaluation Form that is completed at admission. The resident Individual Service Plan will be reviewed for reports of occurrences of R-REM, falls, incidents, and injuries and other covariate information that develop from 3 months prior to baseline through the study end.

#### Accident/incident reports

The New York State Department of Health mandates electronic transfer of accident and incident reports to regional offices as part of licensing standards for ALRs statewide. Federal regulations require the reporting of alleged violations of abuse, mistreatment, and neglect, including injuries of unknown origin to the facility administrator and in accordance with state law. The reports will be reviewed for potential incidents of R-REM and falls. Data will be collected from 3 months prior to the study through the end of data collection. Each incident report reviewed for resident falls/accidents/injuries may also contain evidence of R-REM.

#### Environmental assessment

In addition, physical environment is assessed (direct assessment at local site): The modified Therapeutic Environment Screening Scale (TESS) [46] will be used by research staff to measure the physical environment. The TESS includes 12 domains: unit autonomy, exit control, maintenance, cleanliness, safety, lighting, space/seating, physical appearance/homelikeness, access to outdoors, orientation and cuing, privacy, and noise, and a scale measuring quality. These data will be collected for descriptive purposes and for possible inclusion in hierarchical linear models. Facility and unit characteristics will also be collected.

### Plans to promote participant retention and complete follow-up {18b}

Based on our experience in the conduct of many studies with residents in long term care facilities, we expect 15% attrition at 6 months and up to 40% at 12 months follow-up, mainly related to discharge, hospitalizations, and death. These percentages have been considered in the power calculations. All enrolled individuals who still reside at the facility at the follow-up visits will be invited to participate at follow-up at a convenient time for the respondent. Multiple attempts will be made to interview these residents.

### Data management {19}

#### Data entry and quality

All screening and evaluation data will be collected using a computer-assisted personal interview (CAPI) system. This method provides accuracy in data collection, because the system does not accept out-of-range values, and does not allow for deviation from prescribed skip patterns. The DCC will create scoring and cleaning programs for scales within instruments. Although the CAPI and data entry systems should not allow these types of errors, the cleaning programs serve to double check the accuracy of the data. Periodically, the data manager will review all data for duplicate records, illogical collection dates or times of interview, outlier and out of range values, and illogical contingencies using program syntax created for each data file. After any corrections are made, items distributions will be reviewed to make sure no anomalies remain. In addition, the project coordinator periodically reviews entire files as a quality assurance measure.

#### Data storage, data safety, and security

All laptops used for data collection and office based desktop computers are password protected and Bitlocker encrypted. Laptops will not leave the facility. Laptops with encrypted data are stored in a locked cabinet at the facility and not removed until study end. They are dedicated to the project and not connected to the Internet. Log sheets will also be kept in a locked onsite storage area.

At the DCC, electronic data are backed up daily or weekly to a backup server depending upon the receipt of data. Additional backup external hard drives are stored in a fireproof safe. Protected Health Information (PHI) is confined to a secure device that is not connected to the Internet. All computers are password protected and the whole drive is encrypted with Bitlocker encryption. They are on a non-routable LAN network. No file and database servers are accessible to the public through the Internet. A hardware-based firewall device protects the network system against hackers and any unauthorized Internet access. Spam and email filtering is built-in within the firewall device. The anti-virus software McAfee Anti-Virus protects the network from threats of viruses, worms, and Trojan horses and other malwares contained in email attachments and also from files downloaded through the Internet. Through “push-technology,” this anti-virus software is automatically updated for all virus definitions and other updates.

### Confidentiality {27}

The field coordinator will oversee the onsite assignment of individual IDs. These ID numbers will not contain PHI (e.g., social security number, medical records number) The list of assigned IDs with minimal PHI (name, date of birth, and date of admission) is maintained in a locked cabinet at an on-site location during the course of data collection and later transferred to a secure cabinet at the DCC. Data collection files will be identified only with coded IDs. In compliance with HIPAA, individual participant confidentiality will be assured using ID codes throughout data processing and analyses. Additionally, none of the analyses will permit identification of any individual by name. The interviewers will be aware of the linkage between individual and ID numbers. At the DCC, individual participants will be known only by their ID numbers, which will be used as the basis for communication with the interviewers in the event of data anomalies. The clinical/research barrier will remained intact, in that it is not necessary for any of the data processing staff to be familiar with the identity of the participants. No PHI will be stored in devices linked to the Internet.

### Plans for collection, laboratory evaluation, and storage of biological specimens for genetic or molecular analysis in this trial/future use {33}

Not applicable, there are no biological specimens collected.

## Statistical methods

### Statistical methods for primary and secondary outcomes {20a}

#### Analyses for the primary hypothesis for the distal outcome

The primary analyses will examine, on an ITT basis, the differences between the usual care and intervention groups in the reduction of falls/accidents/injuries over time. Other approaches, e.g., the analysis of the “as treated” sample can result in biased estimates of the causal treatment effect due to compromised random assignment. Our primary approach to analyses is guided by our own experience [47] and reviews [48, 49]. Depending on the level of missing data, analytic strategies (see below) can be used to address non-compliance, e.g., [50]. Based on prior trials that we have coordinated, sampled from this population, 1 year attrition is between 30 and 40%; thus, for 6-month data (projected attrition of 15%), the ITT analyses of all subjects will be feasible. Longitudinal analyses will include those with at least baseline and one follow-up. Sensitivity analysis will be performed using multiple imputation under different assumptions for missing data.

Because randomization is at the facility rather than individual level, imbalance may be observed on baseline variables. Preliminary analyses will be performed to determine whether the groups are balanced. Two-tailed tests of significance will be performed. Binomial tests are to be conducted on dichotomous variables, Poisson tests on nonbinomial (e.g., count) data, and *t* tests on ordinal data, adjusting standard errors for clustered data within facilities; *p* values are reported because the design does not permit randomization at the level of the individual.

##### Effect on likelihood of falls/injuries/accident reduction

Statistical methods appropriate for clustered data will be used in the primary analysis to compare the groups with respect to the binary outcome. Generalized mixed effects models (MEMs) will be used to test the hypothesis that those assigned to the intervention will experience a significantly higher rate of fall/accident/injury reduction as contrasted with usual care. The outcome will be modeled as a function of intervention, time, and their interaction and controlling for the baseline falls. To account for the clustered nature of the outcomes, the models will include random effects for facilities, units, and subjects. The models will be fit using mixed effects logistic regression as implemented in SAS Proc Glimmix. The appropriate covariance structure will be determined. The primary analysis will be supplemented with an exploration of the effect of baseline subject, facility, and unit characteristics on the intervention effect. These results will inform generalizability of intervention effects.

#### Analyses for secondary outcomes

The proposed secondary outcomes are affect, measured by the FTQ and the NIH PROMIS depression measure [51, 52], and behavior. The reliability estimates of the outcomes are typically at least 0.90. Methods for analyses of treatment effects in pre-post clinical trials, in the context of missing data, have been compared [53–58]. The primary proposed analyses will use MEMs, and a full information likelihood approach, with sensitivity analyses using generalized estimating equations (GEE).

##### Statistical models

The primary endpoint analysis for comparison between the two groups with respect to various outcomes will be based on models for the outcome at 6 and 12 months as a function of the intervention condition, adjusting for the baseline value of the outcome, i.e., an ANCOVA-type MEM analysis with intervention as a factor with two levels and baseline value as a covariate. For multiple outcomes (e.g., NIH PROMIS and FTQ), MANCOVA-type MEMs will be used to simultaneously model the multiple outcome variables. An additional factor (domain) denoting the individual outcome variables will be included in the model together with interactions between domain (depression/affect measures) and intervention. A significant interaction term would indicate that the effect of the treatment is different for affect and depression, in that case two treatment effects will be estimated for each outcome. If the interaction is not significant, a model with only main effects for depression/affect and treatment will be fit and the (common) treatment effect will be estimated from this model. In addition to significance testing, we will estimate the treatment effects with 95% CI. The MEMs allow modeling the correlation between the variables assessed on the same subject and that between subjects from the same units and facilities. Group differences in total scores will be examined using a linear mixed (fixed and random effects) model for effect estimation.

The use of SAS Proc MIXED will allow for the possible group heterogeneity in residual variances that may require modeling to satisfy model assumptions and improve model fit and the modeling of the covariance structure. In secondary analyses, we will examine within subjects change over time. We did not perform power calculations for the secondary outcomes because the most stringent power requirements are for the primary outcome. However, we will be able to detect relatively small effect sizes, given the sample size.

Examination of the exploratory potential mediating effects of behavior on the relationship between the R-REM intervention and the falls/injuries/accident outcome will be examined using mediation analyses. Although recent evidence from Monte Carlo studies support simple joint significance tests of the mediating path coefficients [59–61], also examined will be other formal tests of mediation effects [62, 63].

Based on prior experience with the outcomes, it is not expected that transformations will be necessary. Baseline variables will be examined by study arm; however, no *p* values will be provided, and covariates (other than baseline values) are not proposed for inclusion in the main analyses of treatment effects.

##### Examination of potential region differences

Data from regions (upstate and downstate) will be combined and each outcome (e.g., depression, affect) modeled as a function of two factors (i) intervention, a factor with 2 levels and (ii) region, a factor with 2 levels, and the interaction between (i) and (ii). MEMs will be used for each outcome, e.g., depression, and likelihood ratio tests (*χ*^2^) used to test for significance of the interaction term. The study is not powered to detect meaningful regional differences in treatment effect; therefore, we will use *α* = 0.15 as a criterion and explore if regional differences are related to differences in intervention effects.

### Interim analyses {21b}

There are no plans for interim analysis or predetermined stopping guidelines.

### Methods for additional analyses (e.g., subgroup analyses) {20b}

#### Heterogeneity of treatment effects (HTE)

Because there is interest in examining subgroups to determine for which individuals interventions may be effective, we will perform descriptive HTE with potential effect modifiers as interaction terms. HTE will be examined for subgroups, e.g., those with and without cognitive impairment.

### Methods in analysis to handle protocol non-adherence and any statistical methods to handle missing data {20c}

Examination of baseline differences on key variables between completers and those lost-to-follow-up will be conducted to inform about the nature of the missing data. The ITT analyses performed using MEMs will permit all individuals with at least one post-baseline observation to be included.

Depending on the level of missing data, analytic strategies can be used to address non-compliance, e.g., [50]. For the sustainability analyses, analyses will be of those with at least baseline and one follow-up. Sensitivity analysis will be performed using multiple imputation under different assumptions for missing data. Examination of baseline differences on key variables between completers and those lost-to-follow-up will be conducted in order to inform about the nature of any missing longitudinal data. Methods of examining missing data, e.g., propensity scores, inverse probability, EM algorithm, and multiple imputation sensitivity analyses will be considered. If substantial missing outcome data are observed, we will use a specific imputation approach, e.g., Markov Chain Monte Carlo procedures [64], depending on the amount and pattern of missing data. SAS Proc Multiple Imputation and MIAnalyze will be used. We will perform joint simultaneous imputation; at least 50 multiple imputations will be generated and PROC MIAnalyze will be used to combine the results and estimate the log odds, adjusted standard errors, and significance.

### Plans to give access to the full protocol, participant level-data, and statistical code {31c}

There are no such plans at this time.

## Oversight and monitoring

### Composition of the coordinating center and trial steering committee {5d}

The DCC at the RD-HHAR is responsible for oversight of data collection and analyses.

### Composition of the data monitoring committee and its role and reporting structure {21a}

The Data Safety and Monitoring Board (DSMB) or data monitoring committee (DMC) is composed of five members including a chair, clinician/safety officer, and a biostatistician. The NIA project officer will also attend DSMB meetings. The DSMB responsibilities include review the research protocol, informed consent documents, and plans for data safety and monitoring; evaluate the progress of the trial, including periodic assessments of recruitment, accrual and retention, participant risk versus benefit, performance of the trial sites, and other factors that can affect study outcome; review study performance, make recommendations, and assist in the resolution of problems reported by the principal investigator; protect the safety of the study participants; report to NIA on the safety and progress of the trial; and ensure the confidentiality of the study data and the results of monitoring. The board acts independently from the study sponsor.

### Adverse event reporting and harms {22}

All adverse events (AEs) and severe adverse events (SAEs) will be reported by the interviewers to the Project Manager who will update the Adverse Events Reporting Form and alert the PI.

Given that the study intervention is staff training, and there are no physical components to the participant evaluation (e.g., no blood draws or physical examinations), it is expected that no AE or SAEs will be related to study participation. Given that this is an older study population, SAEs (e.g., hospitalizations, death) are expected to occur, with no relationship to study participation. Thus, per the NIA sample SAE/AE Process flow diagram, these events are not unexpected and will be reported to the DSMB at annual teleconferences. However, in the unlikely event that an AE or SAE is determined to be definitely, probably, or possibly related to the study, it will be reported as follows: SAEs will be reported by the PI to the DSMB, NIA, and IRB within 24 h of the event being reported to the investigator; and AEs will be similarly reported within 2 weeks of the event. Although there will be multiple assisted living facilities enrolled, it is unlikely that these participating sites will have their own IRBs; thus, there will not be site-specific IRB notification beyond that to the Weill Cornell Medical Center and the Hebrew Home at Riverdale IRBs.

### Frequency and plans for auditing trial conduct {23}

There are no specific plans for auditing trial conduct.

### Plans for communicating important protocol amendments to relevant parties (e.g., trial participants, ethical committees) {25}

Any protocol amendments will be presented first to the DSMB for approval. If approved, plans will be presented to the IRB.

### Dissemination plans {31a}

Study outcomes will be presented in manuscripts in relevant peer-reviewed journals and at scientific meetings. Depending on the wishes of the facility administrators, there may be a meeting for participating facilities staff to present study findings.

## Discussion

Given the movement toward alternative congregate living arrangements for older individuals with significant comorbidities, including cognitive impairment; it is critical to enhance staff knowledge related to recognition, reporting, and treatment of resident-to-resident negative interactions in such settings to increase resident safety and quality of life. This project is the first to use our R-REM case finding instrument in ALRs, as well as the first integration of a training program and intervention for ALR staff to enhance identification and intervention with respect to episodes of R-REM. The primary study hypotheses are increased staff knowledge after training; increased reporting of R-REM in the intervention as compared to the usual care group; and the frequency of falls, accidents, and injuries will decrease in the intervention group, relative to the usual care group. These findings would be consistent with the results of the nursing home evaluation. The long-term goals of this research are to garner information about R-REM in ALRs that will serve as the basis for additional interventions to prevent it, or avert undesirable associated outcomes when it is unavoidable.

Strengths of this work include its novel case finding methodology, the integration of qualitative and quantitative methods for event description, and the collaboration of a diverse research group uniquely qualified to conduct this work. However, potential challenges relate to site recruitment. The research team has extensive experience conducting intervention research in LTSS settings and successfully implemented and evaluated a similar R-REM protocol in nursing homes.

## Trial status

Protocol version 1.0 dated December 26, 2017. Recruitment began on June 22, 2018, and is expected to be complete by April 1, 2021.

## Abbreviations

AEs: Adverse events
ALRs: Assisted living residences
BRDS: Behavior Recognition and Documentation Sheet
CAPI: Computer-assisted personal interview
CAREDIAG: Care Dementia Diagnostic Scale
DCC: Data coordinating center
DSMB: Data Safety and Monitoring Board
FTQ: Feeling Tone Questionnaire
HTE: Heterogeneity of treatment effects
INCARE: Institutional Comprehensive Assessment and Referral Evaluation
IRB: Institutional Review Board
ITT: Intent-to-treat
LTSS: Long-term services and support
MEMs: Mixed effects models
NIA: National Institute on Aging
PADL: Performance Activities of Daily Living
PHI: Protected Health Information
PROMIS: Patient Reported Outcomes Measurement Information System
RD-HHAR: Research Division, Hebrew Home at Riverdale
R-REM: Resident-to-resident elder mistreatment
SAEs: Severe adverse events
TESS: Therapeutic Environment Screening Scale
